# High-Resolution Microscope-Mode Secondary Ion Mass Spectrometry Imaging

**DOI:** 10.1021/acs.analchem.5c07789

**Published:** 2026-02-24

**Authors:** Yifeng Jia, Maria Elena Castellani, Kieran Cheung, Yuting Su, Michael Burt, Paul Blenkinsopp, Felicia M. Green, Mark Brouard

**Affiliations:** † The Department of Chemistry, The Chemistry Research Laboratory, 6396 The University of Oxford, 12 Mansfield Road, Oxford OX1 3TA, United Kingdom; ‡ 590430 Rosalind Franklin Institute, Harwell Campus, Didcot OX11 0QX, United Kingdom; § Ionoptika Ltd., B6 Millbrook Close, Chandler’s Ford, Hampshire SO53 4BZ, United Kingdom; ∥ Department of Chemistry, 538751 Trent University, 1600 West Bank Drive, Peterborough, Ontario K9L 0G2, Canada

## Abstract

We report the development of a secondary ion mass spectrometry (SIMS) microscope-mode imaging instrument suitable for a wide range of applications in which high throughput is an advantage. By coupling time-of-flight mass spectrometry with pulsed ion extraction methods, the instrument can provide mass resolutions of *m*/Δ*m* ∼ 2000 across a mass range of *m*/*z* ≳ 1000. We show that the use of an ion imaging detector with a fast scintillator screen, yielding time-of-flight time resolutions of a few nanoseconds, improves the mass resolution further to *m*/Δ*m* ∼ 6900. Spatial resolutions of better than 5 μm were also obtained under optimum conditions. We demonstrate the capability of the instrument by imaging atomic and molecular ion species in mouse brain tissue sections, capturing a range of biologically relevant ions, including phospholipid and amino acid fragments, over millimeter length scales within minutes.

## Graphical abstract

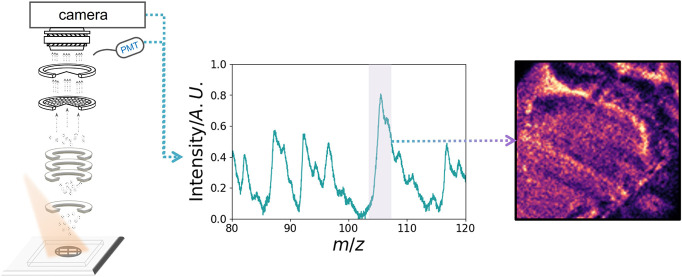

## Introduction

Understanding the chemical complexity of tissues and organs at the cellular level offers immense potential for biology, pharmacology, and medicine.^1–3^ As a label-free technique, mass spectrometry plays a vital role in the qualitative and quantitative analysis of biological chemical composition.^4–6^ By integrating mass spectrometry with imaging capability, mass spectrometry imaging (MSI) enables precise visualization of the spatial distribution of chemical species within samples, enabling breakthroughs to be made in material sciences^7–11^ and biology.^12–17^

MSI strategies can broadly be categorized into two principal modes:^18–20^ microprobe mode^21^ and microscope mode.^22,23^ At present, microprobe mode MSI remains the most widely adopted approach. The commonly employed microprobe mode techniques include matrix-assisted laser desorption ionization (MALDI), secondary ion mass spectrometry (SIMS), and desorption electrospray ionization (DESI),^19^ each offering distinct advantages in terms of spatial resolution, ionization efficiency, sample handling and molecular coverage. In the microprobe mode configuration, a focused primary ion or laser beam sequentially scans the sample surface, and secondary ion signals are recorded on a pixel-by-pixel basis. Thus, while microprobe mode MSI can deliver very high mass resolution, it can also be very time-consuming when acquiring large-area images at spatial resolutions below 10 μm.^24^

Microscope mode MSI^22,23^ is an alternative approach offering a combination of high mass and spatial resolution with relatively high throughput, e.g., the ability to image millimeter scale samples with isotopic mass resolution and sub-5 μm spatial resolution in a matter of minutes. It can be expected that a typical microscope mode time-of-flight (ToF) imaging instrument will have image acquisition rates ten to a hundred times higher compared with those of a typical microprobe mode ToF instrument operating at the same repetition rate and recording images of comparable signal-to-noise and spatial resolution. Unlike microprobe mode, the microscope mode approach enables imaging over larger areas by employing defocused laser or primary ion beams coupled with fast position-sensitive detectors. The latter include delay line detectors^25–27^ and complementary metal oxide semiconductor (CMOS) devices,^28^ such as the pixel imaging mass spectrometry (PImMS) camera^29–32^ and TimePix-based cameras.^33–36^ Demonstrating this potential, Green et al. have recently shown that using SIMS it is possible to defocus the primary ion beam to cover an area with a diameter of ∼2 mm, while maintaining a spatial resolution of ∼20 μm.^37^ However, further development of microscope mode MSI requires improvements to be made in both spatial and mass resolution, while maintaining the benefits of relatively large sample size and high throughput.

The ability to perform microscope mode MSI rests on the precise mapping of the original spatial information on the nascent secondary ions onto the detector, making the ToF technique the preferred option for mass analysis. Compared with Orbitrap^38–40^ and Fourier Transform Ion Cyclotron Resonance (FT-ICR)^41–43^ methods, ToF mass spectrometry (ToF-MS) typically has a lower mass resolution, which potentially limits its broader adoption for MSI. Previous work using microscope mode imaging ToF-MS in its simplest form,^31,34,37,44^ achieved mass resolutions of a few hundred at *m*/*z* ∼ 500. Progress in pulsed ion extraction techniques has led to significant improvements in mass resolution and mass range, particularly for MALDI MSI.^32,45^ Noteworthy in the present context is the post extraction differential acceleration (PEDA) method, initially developed and introduced to MALDI MSI by Aoki et al.^45^ and later enhanced and applied to MALDI MSI by Winter et al.^46^ and Guo et al.^47,48^ Coupling these methods with ToF reflectron imaging techniques,^49^ or the multiturn electrostatic analyzer approach of Aoki and Toyoda,^50^ point the way to further promising avenues for overcoming these resolution limitations.

Here we show that by combining microscope mode SIMS MSI with time-variable PEDA, significant improvements can be made in mass resolution, while preserving high spatial resolution and high throughput over a wide mass window. We illustrated the potential of the instrument by application to the imaging of a biological tissue sample.

## Experimental Method

### Instrument and Data Analysis Procedures

The microscope mode SIMS MSI instrument employed in this work has been described previously,^37^ so only a brief description is given here. It comprised a modified SIMS system, featuring a $C_{60}^{+}$ ion beam (IOG C60-40S, Ionoptika Ltd.) coupled with a linear ToF mass analyzer. Secondary ions generated at the sample surface were extracted and focused using a simple ion lens assembly, comprising the repeller stage, on which the sample is mounted, the extractor, and an einzel lens, details of which have been described previously.^37^ Single^45,46^ and time-variable PEDA^48^ were employed to improve the time-focusing and hence mass resolution of the instrument (see below and Supporting Information (SI) Section S1). Mass analysis was conducted at pressures of ≤2 × 10^–8^ mbar. The uniformity of the defocused $C_{60}^{+}$ primary beam is an important consideration for microscope mode IMS, which has been discussed and demonstrated in our previous work.^37^

The extraction, detection and analysis procedures are illustrated in Figure 1. The $C_{60}^{+}$ primary ion beam was typically operated at an energy of 40 keV. The primary ion beam had an initial pulse width of 100 ns, which was temporally focused (bunched) to around 3 ns to improve time resolution. The primary ion beam, defocused to a diameter of ∼2 mm,^37^ was optimized for a continuous ion beam current of 700–800 pA at a source temperature of 380 °C. A trigger pulse from the $C_{60}^{+}$ primary ion beam was used as the time zero (*t*_0_) for a delay generator, which then triggered the buncher, the PEDA voltage pulsing, the secondary ion deflector, and various detectors, as detailed below.

**Figure 1:**
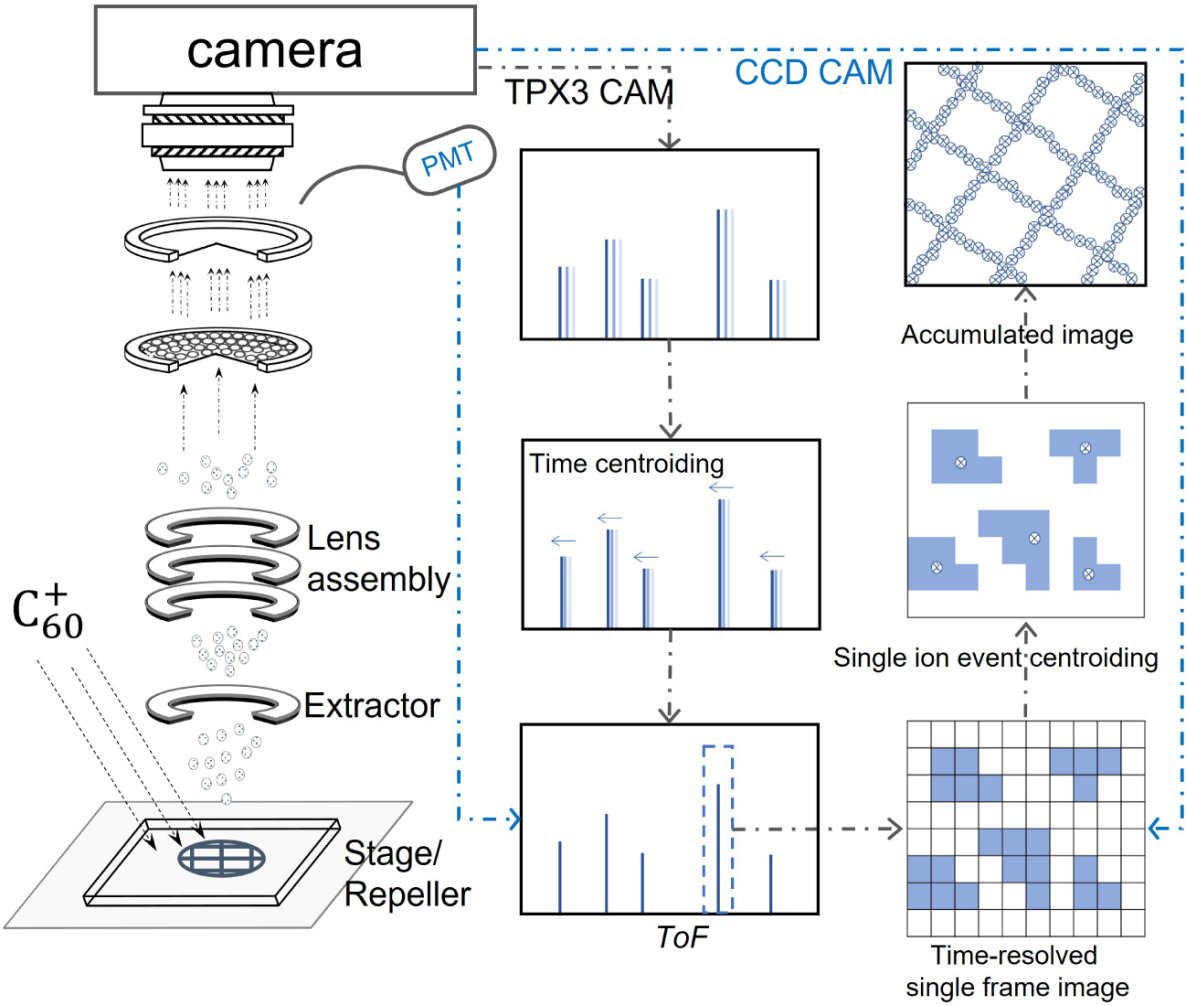
A schematic of the experimental and data analysis procedures are shown in the left and right panels, respectively. Time-of-flight (ToF) spectra were acquired using an MCP detector coupled to a PMT and a TimePix3-based camera, while ion images were captured on a shot-by-shot basis either by a CCD or the TimePix3-based camera. The TimePix3-based system enables the detection of individual ion events with high spatial and temporal resolution, recording each ion’s position and time of arrival. In contrast, the CCD camera is gated over a particular mass peak in the ToF spectrum, and therefore it only provides two-dimensional spatial information for a specific mass peak. Therefore, for CCD-acquired images, only the spatial coordinates are centroided.^51^

Different detection and data acquisition systems were employed depending on the application, including a photomultiplier tube (PMT), an intensified charge coupled device (CCD) camera, or a TimePix3-based camera,^35^ as shown in Figure 1. The PMT and CCD camera were used in tandem as a detection assembly. The PMT was used to acquire the ToF spectra, with each spectrum averaged over 1,000 pulses to enhance the signal-to-noise ratio. Concurrently, the CCD camera was used to acquire mass-gated images for specific ions, providing two-dimensional spatial distributions for selected ion masses. The intensified CCD camera images were obtained at an image acquisition rate of 5 Hz. The TimePix3-based camera was employed to record both the spatial distribution and ToF information simultaneously for all masses following each $C_{60}^{+}$ pulse.^35^ Although the instrument could be run at $C_{60}^{+}$ repetition rates exceeding 1000 Hz, in practice, due to data transfer rate constraints, the TimePix3-based camera was typically operated at 100 Hz, yielding a total acquisition time of around 10–15 min per data set to obtain a reasonable signal-to-noise.

Further data processing was employed to enhance the time and spatial resolution of the ion signals, with images generated in both raw and centroided formats (see SI Section S2). Individual ions impacting on the MCP/phosphor screen detector result in flashes of light on the phosphor which are detected by the CCD or TimePix3-based cameras across multiple pixels, referred to as a cluster. For the CCD-based imaging, individual ion impacts were centroided to a single pixel to enhance spatial resolution.^32,47^ In the case of the TimePix3-based camera, which records the time-stamp of individual ion events on a pixel-by-pixel basis, centroiding was applied to both the ToF and spatial coordinates. Centroiding in the ToF coordinate ensures that the time of the earliest pixel to trigger in a given ion event cluster is taken as the time-stamp of the ion event.^32,35^ Additional corrections were also made to further enhance the temporal resolution of the acquired ToF spectra using the time-over-threshold (ToT) information provided by TimePix3.^35,52,53^

Spatial resolution analysis was performed by extracting intensity profiles across selected grid regions. The spatial resolution was quantified using two methods: one based on curve fitting, and the other employing the standard 20%–80% criterion^32,34,37,46–48^ which measures the width across the rising edge of each intensity peak corresponding to the grid features (see SI Section S2).

### Sample Preparation

To evaluate the performance of the SIMS system, dye samples with grid patterns were employed. For mass and spatial resolution characterization, a series of laser dyes (Auramine O, Rhodamine B, Rhodamine 640, Exalite 404, Exalite 428) and Irganox 1010 were uniformly deposited using a home-built electro-sprayer setup. The laser dye solutions were electro-sprayed onto 25 × 25 mm^2^ indium tin oxide (ITO) coated glass slides or silicon wafers (chips 10 × 10 × 0.5 mm^3^) overlaid with nickel meshes (140 μm pitch) and transmission electron microscope (TEM) support grids with 50/100 mesh sizes (corresponding to 250–500 μm pitch) to generate well-defined grid patterns.

For biological samples, wild type mouse brain tissue (Mary Lyons Center) was cryo-sectioned coronally to a thickness of 10 μm using a cryo-microtome onto silicon wafers. The tissue sections were stored at −80° C and dehydrated before analysis (see SI Section S3).

### Simulation Methods for Post Extraction Differential Acceleration

The current work used time-variable PEDA,^45,47,48^ to enhance the mass resolution of the instrument by time-focusing the secondary ions in a given mass window along the ToF axis. The time-variable PEDA pulse was applied to the extractor electrode, with both the amplitude and timing of the PEDA pulse influencing the achievable mass resolution and mass range. The voltage ratios between repeller stage, extractor, and einzel lens were critical for the spatial focusing of the ions, and the values used in the experiments were first estimated using simulation.

The ion trajectory simulations were carried out using SIMION^54^ (see SI Section S4). Both the simulations and experiments were performed in positive ion mode, with the repeller stage voltage, *V*_R_, held constant at +8.0 kV. To determine the spatial resolution in a simulation, three groups of ions were initialized at distinct positions on the repeller, separated by a distance, *d*_1_. When these ions reached the detector, their final positions were recorded, yielding a new separation distance, *d*_2_. The instrument magnification (*M*) was then defined as *M* = *d*_2_/*d*_1_. Each ion group formed a spot on the detector with a spatial spread characterized by a standard deviation, which was used to calculate the full-width at half-maximum (fwhm) of the signal intensity on the detector. The spatial resolution was subsequently obtained as the ratio of the fwhm of the detector spot to the magnification, i.e., fwhm/*M*. The calculated spatial resolution as a function of the ion-optic voltage settings was also optimized for different degrees of signal magnification.

Five instrument parameters were optimized in the simulations involving time-variable PEDA: the extractor baseline voltage (*V*_E_), the first extractor pulse voltage (*V*_1_), the second pulse voltage (*V*_2_), which was reached using a time dependent exponential voltage ramp, the lens voltage (*V*_L_), and the trigger delay time of the first pulse (*t*_d_) (see SI Figure S1). Optimization was carried out using a genetic algorithm (GA) procedure, which searches for parameter combinations that optimize a figure of merit and hence maximize instrument performance. The fitness function, *f* = *S*_R_/*M*_R_, combined the spatial (*S*_R_) and mass (*M*_R_) resolution, where a lower spatial resolution and higher mass resolution were desired. Consequently, optimal voltage settings were selected when the GA yields a minimum value of the fitness parameter, *f*, corresponding to a configuration that simultaneously enhances both spatial resolution and mass resolving power.

The simulated voltage settings were verified experimentally by fixing the stage and lens voltages (see Table 1) and scanning the extractor voltage in 10 V steps under static conditions (i.e., without using a PEDA pulse). The experimental voltage at which the ion image with the grid pattern came into sharpest focus defined the optimized setting. The experiments (as shown later) indicated that the inaccuracy of the simulation is less than 20 V, confirming the reliability of the ion trajectory simulations.

**Table 1:** Voltage and Delay Time Settings on the Ion Optics for Static Mode MSI (Top Row) and for PEDA Mode (2nd and 3rd Rows), and Those Employed for Tissue MSI (Bottom Row)^a^

Settings	*t*_d_	*V*_R_	*V*_E_	*V*_1_	*V*_2_	*V*_L_
Static	N/A	8000	5530	N/A	N/A	–15000
PEDA (*m*/*z* 250–600)	380	8000	5530	5870	6870	–14300
PEDA (*m*/*z* 500–1250)	450	8000	5530	5870	6870	–14300
Tissue MSI (*m*/*z* ≤ 200)	180	8000	5530	5870	6870	–14300

a The latter was optimized for the mass window below *m*/*z* 200. The voltages (in volts correspond to the repeller voltage (*V*_R_), the extractor baseline voltage (*V*_E_), the first extractor pulse voltage (*V*_1_), the second pulse voltage (*V*_2_), and the lens voltage (*V*_L_). The parameter *t*_d_ defines the trigger delay time (in nanoseconds) of the PEDA pulse (see text and SI Section S1 for details).

## Results and Discussion

### Time-Variable PEDA Results

Following the optimization of the time-variable PEDA pulse based on the simulation results, the voltages for positive ion detection were set as shown in Table 1, either for the experiments with static ion-optical potentials or for those employing time-variable PEDA.^47,48^ The trigger time, *t*_d_, of the experiment was defined with respect to the firing of the $C_{60}^{+}$ primary ion beam.

The time-variable PEDA pulse is effective when the secondary ions are located in the region between the extractor and the lens. Consequently, the trigger delay and the spatial separation between the extractor and lens predominantly determine the optimized mass range. The optimized mass range corresponds to the range of ion masses that can be stored and accelerated within the region between the extractor and the einzel lens. This range is fundamentally constrained by the physical distances between the repeller, extractor, and lens electrodes. By adjusting the PEDA trigger time it is therefore possible to adjust the mass window over which one optimizes mass and spatial resolution. Two PEDA trigger delays, 380 and 450 ns, were used to optimize the mass resolution across different *m*/*z* ranges, as detailed below.

The top row of panels (i)–(iii) of Figure 2 shows ion images for a ∼2 mm diameter gridded sample comprising various dyes. The pitch of the grids employed was 250 μm. Figure 2a presents the ToF mass spectra associated with the ion images obtained using either a static electric field (red line) or time-variable PEDA (blue line) with a 380 ns trigger delay. Under static extraction conditions (see Table 1), the mass spectrum displays a mass resolution of approximately 500 *m*/Δ*m*. When time-variable PEDA is employed, using a 380 ns trigger delay, the mass resolution is significantly improved, exceeding 2000 *m*/Δ*m* across the mass range *m*/*z* 250–600, and covering a mass window of *m*/*z* ≃ 350. At the same time, the ion images retain a spatial resolution of around 20 μm at a signal magnification of ×11.2, as shown in the top row of images shown in Figure 2.

**Figure 2:**
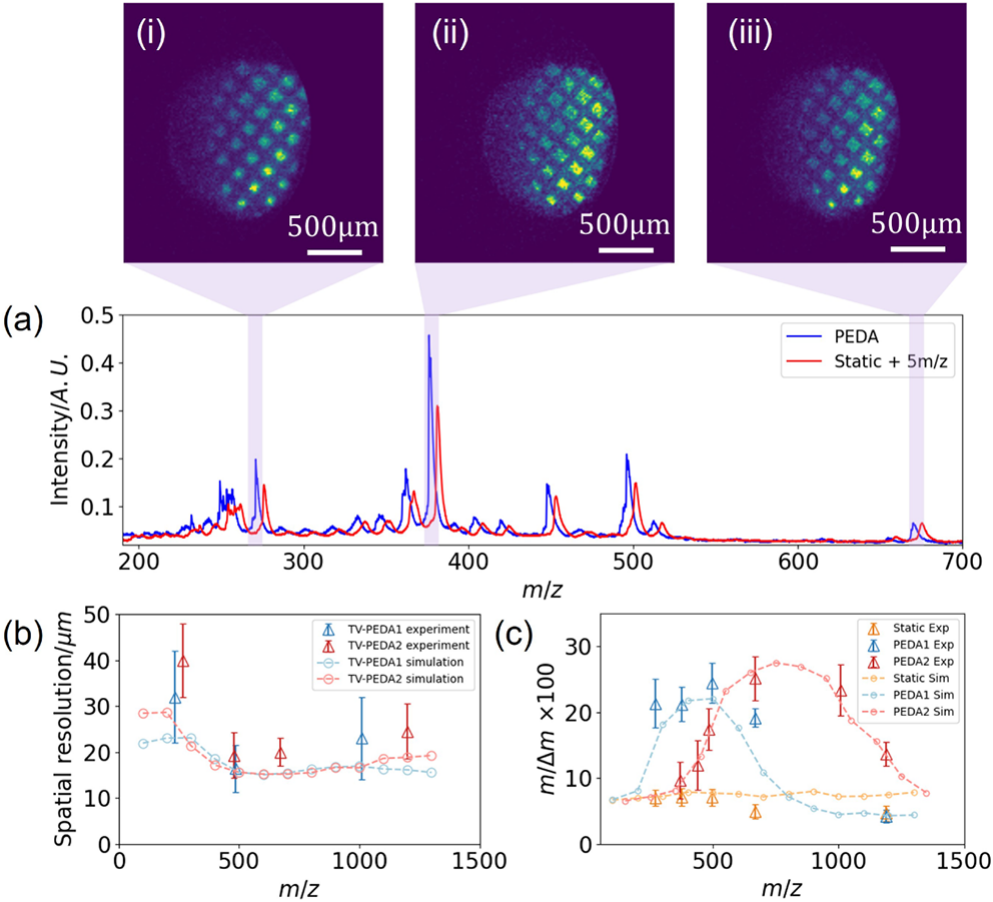
Panels (i)–(iii) on the top row show ion images (top row) for mass peaks correspond to Auramine O, Rhodamine B, and Exalite 404, respectively. Panel (a): the mass spectrum (blue line) obtained using time-variable PEDA,^48^ with the mass spectrum also compared with that obtained employing a static electric field (red line). Note that the static ToF data is shifted 5 mass units (*m*/*z*) to higher mass for clarity. Panel (b) compares the simulated and experimental spatial resolutions obtained with PEDA trigger time-delays of 380 ns (light red and light blue) and 450 ns (dark red and dark red). Panel (c) shows a similar comparison of the mass resolutions obtained with time-variable PEDA at the trigger times of 380 ns (blue) and 450 ns (red). The mass resolutions obtained with static electric fields are also shown in the panel (yellow data).

By adjusting the trigger delay to 450 ns, it was possible to optimize the *m*/*z* 500–1250 mass window. Under these conditions, the mass resolution remained above 2000 *m*/Δ*m*, as shown both from simulation and the experimental data presented in Figure 2c. The corresponding mass spectra are shown in SI Figures S2 and S3. Under these optimized conditions, the spatial resolution is approximately 20 μm (see further discussion below and SI Figures S2 and S3).

Simulations were used to explore further the influence of the various interelectrode gaps on the mass and spatial resolution, and particularly the mass range (see SI Section S4). For example, reducing the distance between the repeller and the extractor leads to a notable enhancement in the mass range. This effect arises because the gap between the repeller and extractor is smaller than that between the extractor and the lens, and primary ion acceleration predominantly occurs within this shorter region. A reduced acceleration distance compresses the spread in ion velocities and consequently narrows the mass dispersion at the point of extraction. As a result, increasing the gap between the extractor and the lens allows a greater number of distinct ion masses to be optimally focused, thereby broadening the effective mass range of the system.

The response time of the detector also limits the mass resolution of the ToF spectrum. The phosphor screen used on the MCP detector employed here was P47, which exhibits a rise time of 6.7 ± 0.2 ns and a decay time of 168 ± 2 ns.^55^ This relatively long decay time can limit the accuracy of full-width at half-maximum (fwhm) measurements of individual mass peaks used to calculate the mass resolving power of the instrument. To reduce the influence of the phosphor time response, we instead used the leading edge of the mass peak (20–80%) to estimate the peak width from which the mass resolution is calculated.^37^ An alternative method to mitigate the effects of the decay tail of P47 is to analyze the mass peaks in derivative mode, as discussed in the SI Section S1. The derivative method yields similar fwhm to those obtain using the leading edge of the raw data.

To further address the limitation imposed by the P47 phosphor screen, we also investigated an alternative scintillator screen (BC-408) with a rise time of ∼1.0 ns and a decay time of ∼2.1 ns.^56^ Mass spectra of the molecular ion of Rhodamine B obtained with P47 and BC-408 were collected, as shown in Figure 3a. The red line corresponds to the signal acquired with P47 under static field conditions, while the purple line represents P47 using time-variable PEDA. Although PEDA significantly sharpens the peak and leading edge of mass peaks, the signal remains broadened owing to the long decay time of the P47 screen. In contrast, the blue line illustrates the mass spectra obtained using the BC-408 scintillator using PEDA. The BC-408 scintillator yields a much higher mass resolution, improving from 2120 ± 315 *m*/Δ*m* (P47) to 6900 ± 400 *m*/Δ*m* (BC-408) (see Figure 3b). These values, calculated from the rise edges, are consistent with the results of simulations. Figure 3c compares the experimental results with those obtained from a simulation of the influence of scintillator rise time on the achievable mass resolution in the current instrument. Unsurprisingly, as the scintillator rise time decreases, the resolution improves accordingly. In the ideal case of zero rise time, the simulated mass resolution reaches up to ∼11,000 *m*/Δ*m*.

**Figure 3:**
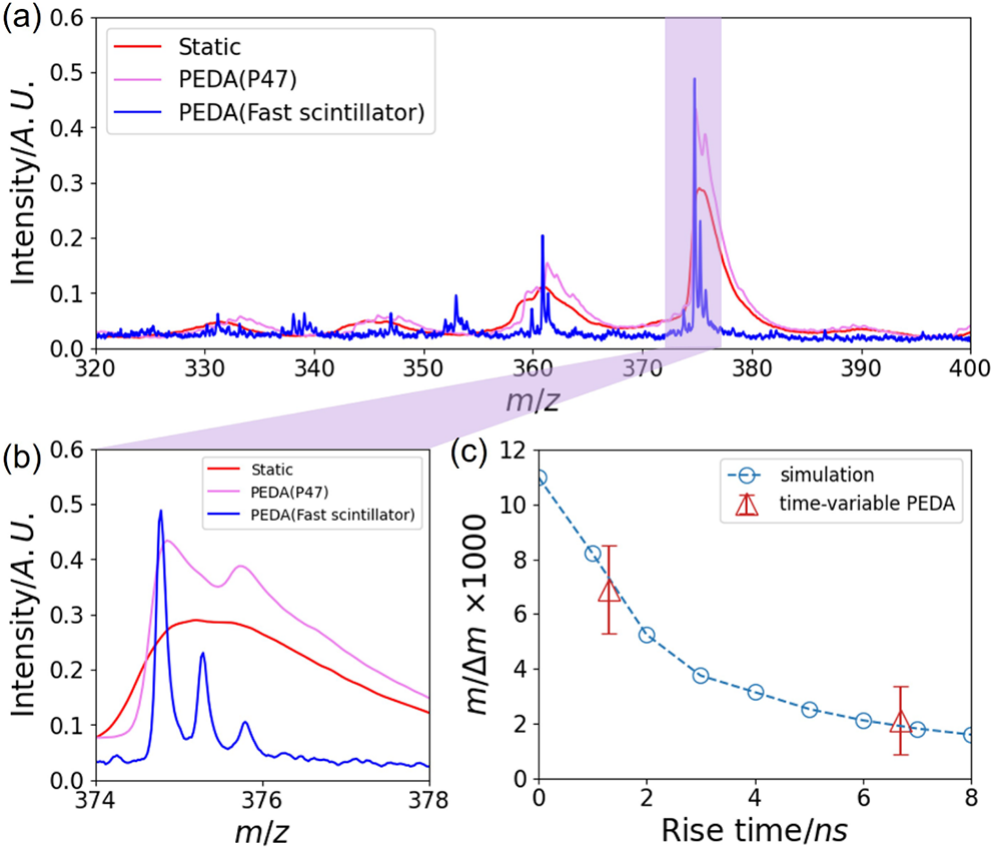
Panel (a) shows the Rhodamine B mass spectra obtained with a static extractor voltage (red), a time-variable PEDA pulse (purple) using a P47 phosphor screen, and the same PEDA pulse employing a BC-408 scintillator (blue). Panel (b) shows an expanded view of the Rhodamine B mass spectrum, highlighting the isotopic pattern of the [M–Cl^–^]^+^ mass peak. Panel (c) compares the experimentally determined mass resolution (measured using MCP detectors employing two different scintillators, P47 or BC-408, coupled to a PMT—red symbols), with the results of the SIMION simulations (blue data), illustrating the effect of detector rise time on the mass resolution.

The full advantages of microscope mode MSI can only be realized using fast ion imaging detection, that enables high mass and spatial resolution data to be obtained concurrently. Here we used the MCP detector with a P47 phosphor screen coupled to the TimePix3-based camera^35^ to capture both the spatial and temporal information for each ion event. The data were collected over 15 min at 100 Hz. Several ion species, including Na^+^, K^+^, In^+^, the Auramine O parent ion ([AO–Cl^–^]^+^), and the Rhodamine B parent ion ([Rho.B–Cl^–^]^+^), were selected to demonstrate the capabilities of the instrument.

The optimized mass spectrum and ion images using the TimePix3-based camera were collected using PEDA with a 380 ns trigger delay, as indicated by the blue line in Figure 4. Compared with the static voltage settings, the application of PEDA produces somewhat sharper and more distinguishable mass peaks. Using the leading edge of the peak, the measured mass resolution with PEDA was 910 ± 60 *m*/Δ*m* at *m*/*z* 367 and 931 ± 78 *m*/Δ*m* at *m*/*z* 437. The TimePix3-based camera operates with a time-stamping precision of 1.5625 ns,^57^ but the time resolution is limited by several factors beyond the time-stamping precision, including the duration of the $C_{60}^{+}$ primary ion beam pulse, the rise-time of phosphor screen, and any jitter in the triggering of the instrument.^35^ In the experiment shown in Figure 4, the TimePix3-based camera was triggered by a light emitting diode (LED), which introduced a time jitter with a standard deviation of around 10 ns, similar to our previous work,^35^ which is the primary limiting factor in the time-response, hence mass resolution, of the current experiment. Despite this limitation, the results serve to further highlight the potential of the TimePix3-based system for rapid and high-resolution mass spectrometry imaging, with acquisition times reduced to the minute time scale.

**Figure 4:**
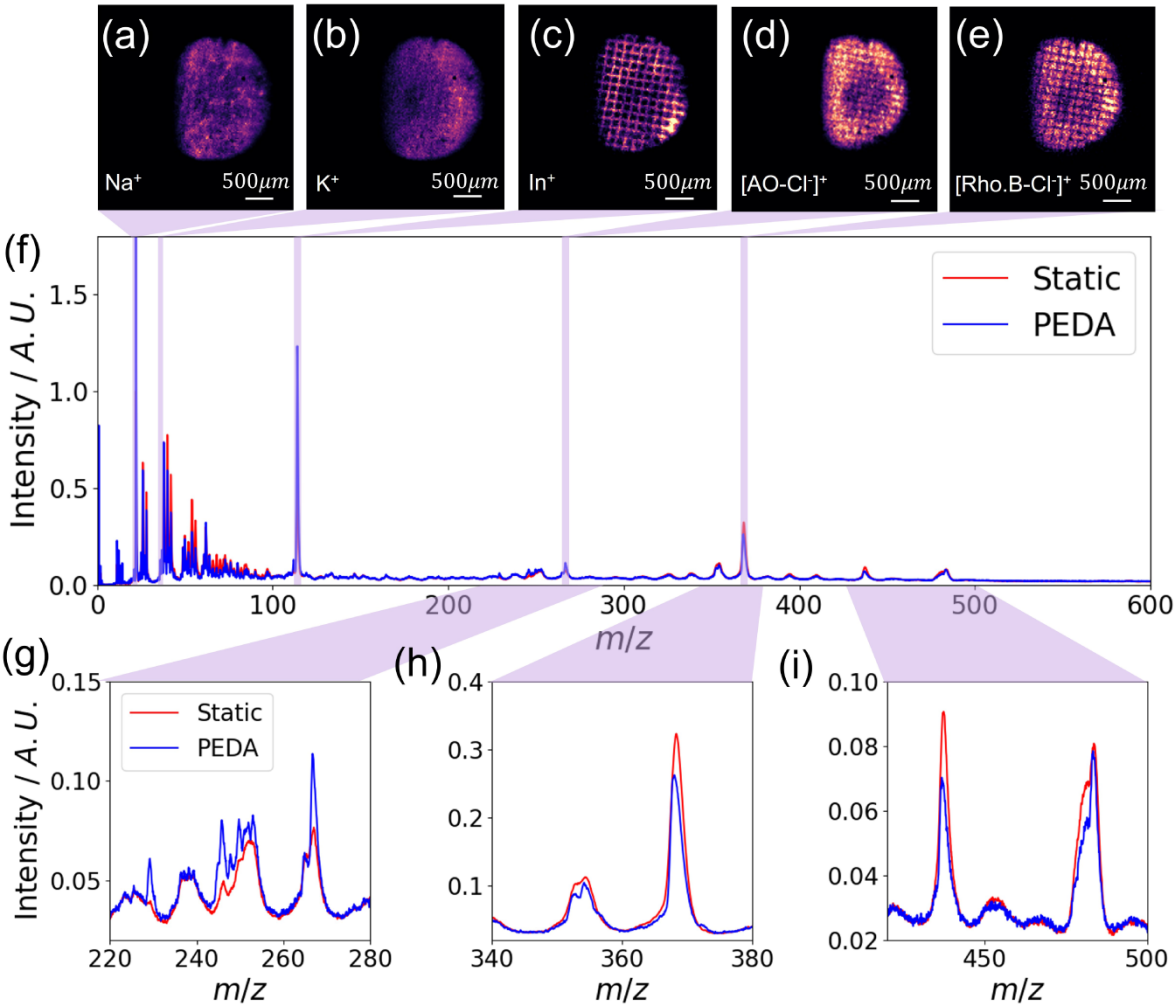
Top: spatial ion images of Na^+^ (a), K^+^ (b), In^+^ (c), [AO–Cl^–^]^+^ (d) and [Rho.B–Cl^–^]^+^ (e) under PEDA condition, demonstrating the imaging capability of the TimePix3-based camera. Panel (f) shows the mass spectrum obtained from dye sample on an ITO substrate, with different amplified mass ranges (*m*/*z* 220–280, *m*/*z* 340–380, and *m*/*z* 420–500), with static (red) and PEDA (blue) conditions.

### Spatial Resolution Enhancement

To enhance the spatial resolution of the instrument, our experimental approach was guided by the SIMION trajectory simulations. We systematically investigated a range of electrostatic lens configurations to identify the optimal setting that maximizes spatial resolution. While our previous work has demonstrated a spatial resolution of 23 μm based on analysis of individual mass-gated images,^37^ the simulations have revealed the potential to attain spatial resolutions below 2 μm under ideal conditions. Instrumental factors that limit resolution include the number of pixels on the detector, the number of pixels in a cluster corresponding to detection of a single ion event (see the Experimental Method Section), and the contributions of optical aberrations.

The ion images recorded with the TimePix3-based camera, shown in Figure 4a–e indicate a spatial resolution of approximately 25 ± 3 μm. This resolution is primarily limited by the pixel count of the camera (256 × 256 pixels), with the effective signal area covering only about 80 × 80 pixels at a magnification of ×11.2. It is worth noting that future generations of TimePix are likely to have a higher pixel count, and thus will be limited less by this constraint. The impact of the number of pixels on the detector is determined by the image magnification within the ion optical assembly. This is controlled by the einzel lens,^37,58^ which allows adjustment in magnification through changes to the applied voltage. In the data presented in Figure 5, the stage voltage was held constant at +8.0 kV, and the magnification varied from ×11.2 to ×44.8 by tuning the einzel lens voltage from −16 kV to 0.0 kV. The extractor voltage was correspondingly adjusted to match the varying lens voltage settings to maintain the mass resolution.

**Figure 5:**
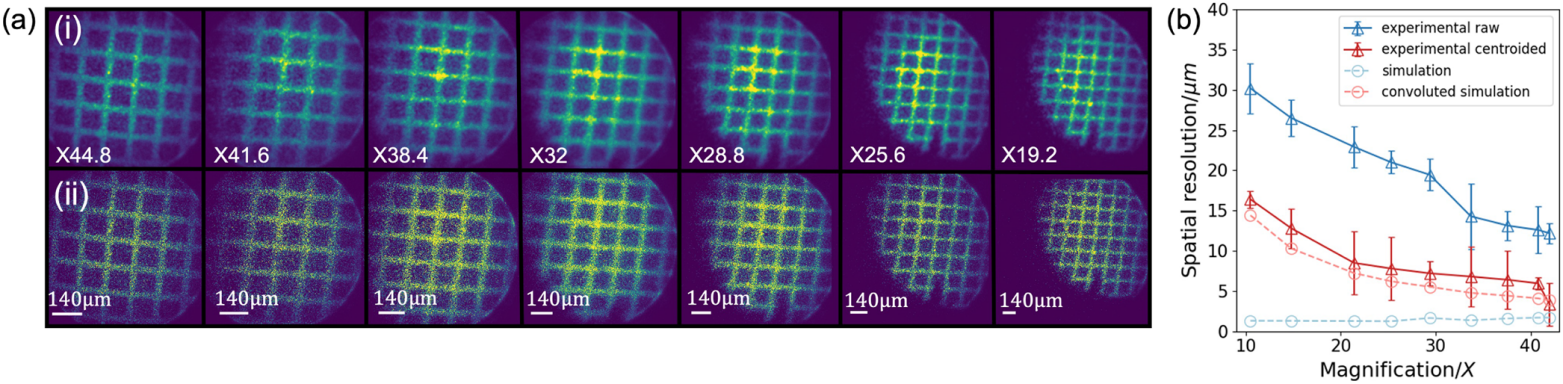
(a) Raw (i) and centroided (ii) In^+^ images at different magnifications. (b) Magnification impact on spatial resolution : this panel illustrates the effect of magnification on spatial resolution. The solid lines correspond to the experimental data from Panels (i) (dark blue) and (ii) (dark red) in Panel (a), representing the raw and centroided spatial resolutions, respectively. The dashed curves show simulation results: the raw simulated spatial resolution (light blue) and the simulated spatial resolution convoluted with the detector pixel size (light red). The pitch of the grid was 140 μm, as indicated.

Raw ion images of In^+^ ions, shown in Figure 5a­(i), were collected using an MCP-P47 and CCD detection system at different magnifications by applying increasing voltages to the einzel lens (see SI Figure S6 for an example of the associated mass spectrum). The CCD camera was used here due to it having a higher number of pixels than TimePix3, leading to a higher spatial resolution. Note that the In^+^ signal comes from the ITO substrate, which is not overlaid by dye sample when covered by the grid during electro-spraying. The corresponding spatial resolutions of these images are indicated by the blue line in Figure 5b. It shows that increasing the image magnification leads to an improvement in spatial resolution. This improvement arises from the fact that higher magnification distributes the ion signal across a greater number of detector pixels. Under optimized conditions, +8.0 kV applied to the repeller, +7.4 kV to the extractor, and 0.0 kV to the lens, a mean spatial resolution of 12.2 ± 1.2 μm was achieved at the highest applied magnification of ×44.8.

Another factor limiting the spatial resolution is the number of detector pixels spanned by a single ion event. This is determined by the cascade of electrons from the MCP channels onto the phosphor or scintillator screen, and the size of the resulting flash of light detected by the camera. To mitigate this effect, a centroiding algorithm was applied to compress the spatial distribution of each ion event by calculating its center of intensity and reassigning it to a single representative pixel.^32^ The process of image centroiding employed here is explained in more detail in SI Section S2. It serves to eliminate background signals and condense target ion events to a single pixel. All images were processed using a centroiding algorithm with a cluster size threshold of 3; that is, ion events with cluster sizes greater than 3 pixels were centroided into a single pixel, with smaller cluster events discounted. Figure 5a presents the ion images (i) before and (ii) after centroiding, demonstrating a substantially sharper appearance with an estimated two to 3-fold improvement in resolution, as demonstrated by comparison of the blue and red lines in Figure 5b. At the highest magnification, the spatial resolution achieved is 3.6 ± 2.1 μm. The experimental spatial resolution remains slightly inferior to the simulated values, partly due to the imperfect description of aberrations in the ion optics. The extraction electrode features a 2 mm central aperture, and simulations suggest that the edge effects of the extraction electric field at this edge induce spherical aberrations, which lead to a slight, but observable, image distortion. A further source of inaccuracy in the simulation may be the velocity distribution of the secondary ions. We have recently directly measured the velocity distributions of particular secondary ions in our instrument,^59^ and the simulations employ these distributions. However, slight unaccounted for variations between the velocity distributions of different secondary ions might be a further factor in leading to a modest discrepancy between the simulations and experiment.

### High-Throughput MSI of Mouse Brain Tissue

The optimized SIMS-MSI instrument was employed to map the spatial distribution of species in a mouse brain tissue section.^14,60–63^ The mouse brain tissue was taken at the infundibulum (IF) and/or median eminence, and the imaging section was chosen in the region that crosses the fronto-parietal cortex (FrPT) and hippocampus (Hipp)^64^ (see SI Section S3 for optical images and details of the region imaged within the brain). Figure 6a presents K^+^ ion images of the selected region acquired at different magnifications (×11.2, ×19.8, and ×44.8). The image data were collected with acquisition times of 5, 10, and 20 min, respectively, using the modified PEDA voltages given in Table 1, which were optimized for the mass window below *m*/*z* 200 where the majority of lipid peaks are found. Note that a larger magnification inherently results in lower signal intensity per pixel. As the magnification increases, an improvement in spatial resolution is observed, resulting in enhanced clarity of the selected region or highlighted region in Figure 6a, and a greater definition of its textural features.

**Figure 6:**
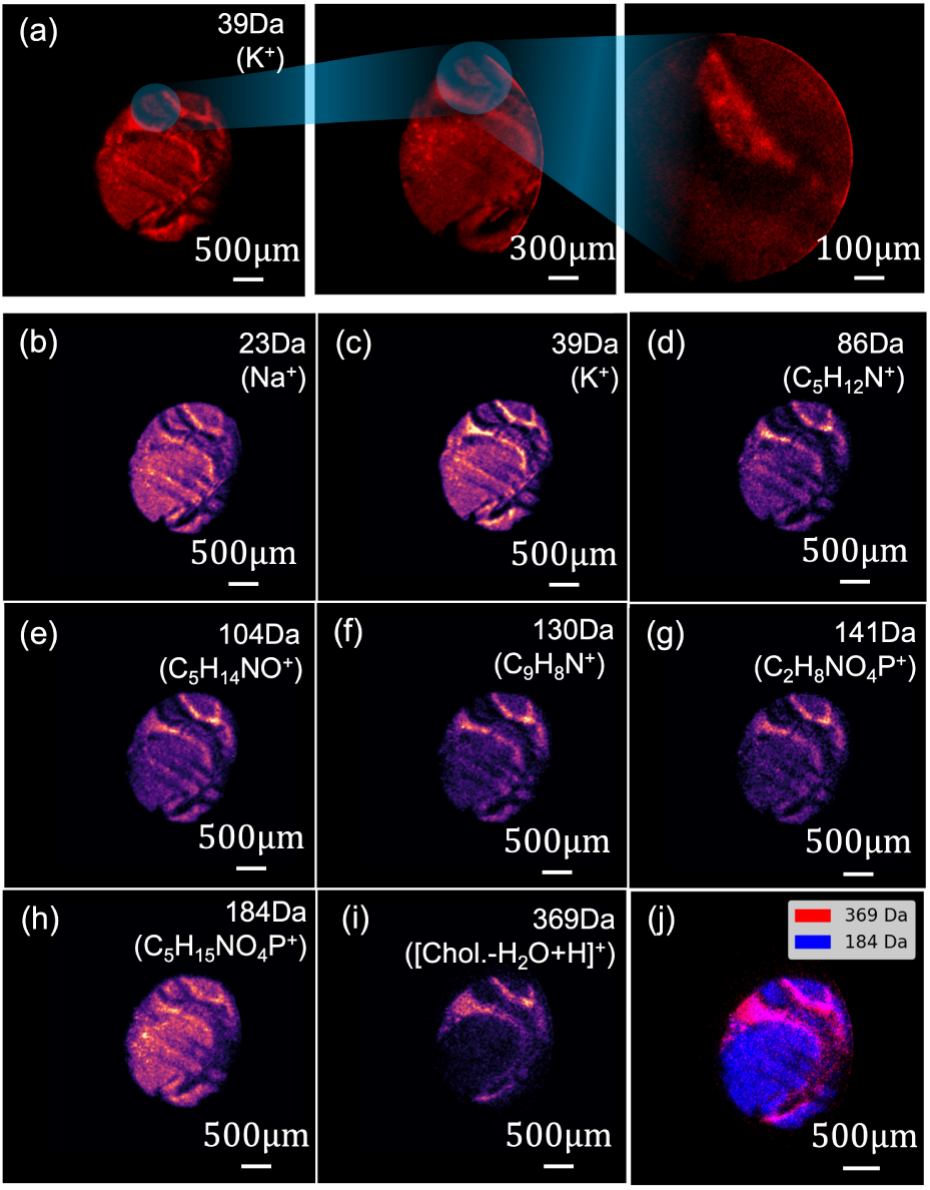
Panel (a): microscope-mode SIMS CCD images of a mouse brain section acquired in 5, 10, and 20 min for low, medium and high image magnification. Because of the different magnifications employed, the spatial resolutions of the K^+^ spatial images range from ∼3 to 12 μm. The low magnification (×11.2) 5 min MSI data are shown in panels (b)–(i) for different inorganic and organic ions, demonstrating the rapid imaging of tissue samples. Panel (j) shows an overlay of the mass *m*/*z* 184 and 369 peaks, highlighting that these species are associated with different areas of the brain (gray and white matter, respectively).

Panels (b–i) of Figure 6 display ion-specific images at a magnification of ×11.2, highlighting the heterogeneous distributions of chemical species. This is illustrated in Panel (j), which shows an overlay of the mass *m*/*z* 184 and 369 peaks, revealing that these species are associated with different areas of the brain (gray and white matter, respectively). The corresponding mass spectrum is shown in SI Figure S7. Potassium (*m*/*z* 39, Panel (c)) and sodium (*m*/*z* 23, Panel (b)) are widespread, with K^+^ showing elevated intensity in the cortex, reflecting its role in neuronal signaling. Distributions of some fragments derived from phospholipids and amino acids are also shown in Figure 6 (also see SI Table S1). C_5_H_14_NO^+^ (*m*/*z* 104) is assigned to choline^65,66^ and C_2_H_8_NO_4_P^+^ (*m*/*z* 141) to a diagnostic fragment for phosphatidylethanolamine.^67,68^ C_5_H_13_NO_3_P^+^ (*m*/*z* 166) and C_5_H_15_NO_4_P^+^ (*m*/*z* 184) are characteristic fragments of phosphocholine-containing lipids.^65^ $C_{27}H_{45}^{+}$ (*m*/*z* 369) is well established as the dehydrated fragment of cholesterol ([M–OH]^+^).^69,70^ Cholesterol is strongly localized to tissue regions corresponding to white matter, whereas phosphocholine is primarily localized to gray matter regions, consistent with Panel (j) of Figure 6, and in qualitative agreement with the reported lipid compositions of brain tissue.^69,71^ Peaks characteristic of amino acids such as C_5_H_12_N^+^ (*m*/*z* 86) assigned to leucine and C_9_H_8_N^+^ (*m*/*z* 130) assigned to tryptophan^65^ are also shown. These are putative assignments; however, the observed distributions of phosphocholine and cholesterol are broadly consistent with previous ToF–SIMS studies.^61,63,69,72–74^

Simulations mentioned above, and discussed in more detail in SI Section S4, suggest that further significant improvements in instrument performance, such as doubling the achievable mass range with mass resolution >10,000, should be achievable by implementing relatively straightforward modifications to the ion optics configuration. Taken together with the results presented in Figure 6, which demonstrate high-resolution molecular imaging, the work presented here underscores the capability of microscope mode $C_{60}^{+}$ SIMS to resolve molecular heterogeneity in brain tissue, offering insights for spatial “omics” studies. This complements our ongoing development of a high-speed MSI instrument for proteomic analysis, providing a potential foundation for mapping complex biomolecular landscapes in neurological disease research.

## Conclusion

This work presents results using a newly developed microscope-mode SIMS imaging instrument. Through the implementation of time-variable pulsed extraction differential acceleration methods, mass resolutions exceeding 2000 *m*/Δ*m* and up to 6900 *m*/Δ*m* have been achieved using an MCP detector coupled with either a P47 phosphor screen or a fast BC-408 scintillator, respectively. By tuning the einzel lens voltage, signal magnification can be increased up to ×44.8, yielding a spatial resolution of better than 5 μm. The performance of the instrument for wide-field, high-resolution mass imaging within minutes is further enhanced by employing a TimePix3-based camera, which enables simultaneous acquisition of ion images and time-of-flight (TOF) spectra at ∼1 kHz repetition rates. This capability is exemplified through application to the spatial mapping of lipids, amino acids and inorganic ions in mouse brain tissue, with both mass specificity and fine spatial detail. Further enhancements in performance can be anticipated, through use of higher energy primary beams,^75^ running at higher repetition rates and with higher beam currents, improved ion-optic design for the secondary ions operating at higher extraction voltages, and developments in fast imaging detector technology.

Taken together, the approach used here represents a significant advancement in microscope-mode SIMS MSI,^37^ and offers an appealing solution for high-throughput spatial “omics”, clinical diagnostics, and large-area tissue-based studies. The instrument could also be very useful for materials and device analysis,^23^ where peak assignments may be simplified and the mass range more limited. The demonstrated performance improvements to, and versatility of, the instrument support its potential for broader adoption in a wide range of translational research.

## Supplementary Material



## Notes

More experimental and simulation data: (1) mass resolution and mass range using electrodynamic PEDA; (2) spatial resolution; (3) mass spectra for frozen mouse brain tissue; (4) simulations of possible instrument improvements (PDF)
