# Supplementary material for: High-Resolution Microscope-Mode Secondary Ion Mass Spectrometry Imaging
Source: Anal Chem. 2026 Feb 24;98(9):7028–37. doi: 10.1021/acs.analchem.5c07789 (PMC12980498; doi:10.1021/acs.analchem.5c07789)
Supplement: Supplementary file 1 [file ac5c07789_si_001.pdf]

Supporting information: High-resolution microscope-mode secondary ion mass spectrometry imaging

Yifeng Jia,† Maria Elena Castellani,†,‡ Kieran Cheung,† Yuting Su,† Michael Burt,†,§ Paul Blenkinsopp,¶ Felicia M. Green,*,‡ and Mark Brouard*,†

†*The Department of Chemistry, the University of Oxford, The Chemistry Research Laboratory, 12 Mansfield Road, Oxford, OX1 3TA, United Kingdom*

‡*Rosalind Franklin Institute, Harwell Campus, OX11 0QX, United Kingdom*

¶*Ionoptika Ltd., B6 Millbrook Close, Chandler's Ford, Hampshire, SO53 4BZ, United Kingdom*

§*Department of Chemistry, Trent University, 1600 West Bank Drive, Peterborough, K9L 0G2, ON, Canada*

E-mail: felicia.green@rfi.ac.uk; mark.brouard@chem.ox.ac.uk

February 17, 2026

Table of contents

- **S1:** Mass resolution and mass range using electrodynamic PEDA.
- **S2:** Spatial resolution.
- **S3:** Mass spectra for frozen mouse brain tissue.
- **S4:** Simulations of possible instrument improvements.

S1: Mass resolution and mass range using electrodynamic PEDA

Before discussing the post extraction differential acceleration (PEDA) technique in more detail, we start by considering the methods used to determine the mass resolution of the instrument. As noted in the main text, this was generally determined from the leading edge of the ToF mass peak (20–80%) in order to minimize the effects of the slow decay of the P47 phosphor screen of the detector. Derivative mass spectra were also used to limit these effects, and help resolve adjacent mass peaks. An example of a derivative mass spectrum is presented in Figure S1. The sample employed comprised a mixture of dyes, including Rhodamine 640, Rhodamine B, Auramine O, and Exalite 428, electrosprayed in a grid pattern onto an ITO-coated slide. In the derivative spectrum, the leading edge of each original mass peak appears as a positive signal, whereas the trailing edge appears as a negative signal. This method yielded very similar full-width-at-half-maximum (FWHM) values to those obtained using the leading edge of the raw spectra, as discussed in the main text.

Turning to the optimization of electrodynamic (time-variable) PEDA, mass spectrometry imaging (MSI) data for the lower mass range (m/z 250–600) were presented in Figures 2 to 4 of the main text, and a summary of the PEDA voltages employed was given in Table 1. The form of the time-variable PEDA voltage pulse applied to the extractor ion optic is illustrated in Figure S1(a).[1,2] As discussed in the main text, time-variable PEDA is only effective within a certain mass range, and adjustments to the PEDA pulse timing are required to study different mass windows. By delaying the PEDA trigger time from 380 ns to 450 ns, the optimised mass range was shifted to cover m/z 500–1200. The same sample as shown in Figure S1 was employed here to quantify the PEDA optimisation for higher masses. As shown in Figure S2 (a) and (d), the Exalite 428 peak exhibited improved time focus when PEDA was applied, yielding a mass resolution of $2300.0 \pm 400\, m/\Delta m$ at m/z 1010. The corresponding Exalite 428 ion image also displayed enhanced focus, achieving a spatial resolution of 23 ± 9 µm.

To further demonstrate the benefits of the PEDA technique, another organic sample,

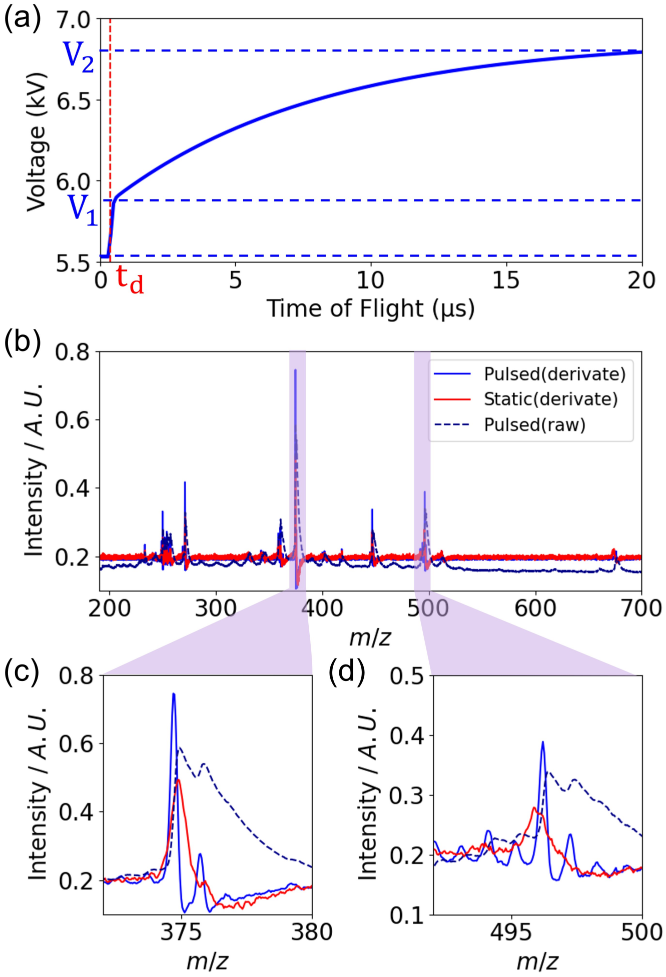

Figure S1: Panel (a): The time dependence of the PEDA pulse used for time-variable PEDA, with PEDA voltages V_1 and V_2 defined. Panel (b) Derivative mass spectrum obtained using PEDA optimised for the mass window between m/z 250–600. The spectrum was collected using a MCP-P47 detector and PMT. The red curve corresponds to the spectrum acquired with static extraction fields, while the blue curve represents the spectrum obtained with PEDA using a trigger delay of 380 ns. Panels (c) and (d) present magnified views of the mass ranges m/z 355–380 and m/z 485–510, respectively. The sample employed comprised a mixture of dyes, including Rhodamine 640, Rhodamine B, Auramine O, and Exalite 428, electrosprayed in a grid pattern onto an ITO-coated slide.

Irganox 1010, was tested. A uniformly coated Irganox 1010 layer was covered with a TEM support grid (mixed mesh with a pitch size of 62–125 µm). The associated mass spectra acquired with (blue) and without (red) PEDA (450 ns trigger delay) are shown in Figure S3 (a), while the corresponding ion image obtained with PEDA is shown in Figure S3 (b). Analysis of the rising edge of the mass peaks yielded a mass resolution of $1370 \pm 180\, m/\Delta m$ at

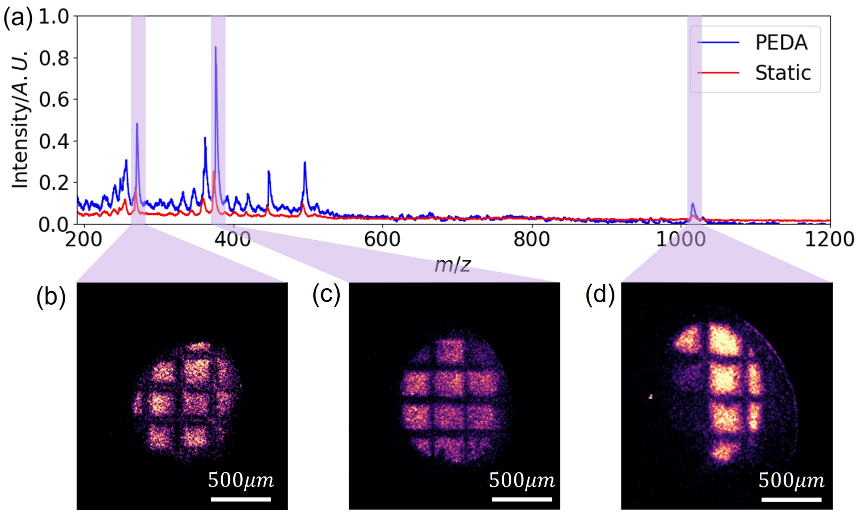

Figure S2: Panel (a) shows the mass spectrum of the mixed dyes, including Rhodamine B, Rhodamine 640, Auramine O, and Exalite 428 which were collected using a PMT. The red curve corresponds to the spectrum acquired under a static extraction field, whereas the blue curve represents the spectrum obtained with PEDA. Bottom panels: spatial ion distributions of Auramine O (b), Rhodamine B (c), and Exalite 428 (d) grids acquired using PEDA with a 450 ns trigger delay. The grid pitch size is 250 µm.

m/z 1189. The spatial resolution measurement is presented in Figure S3 (c). A Gaussian fit was applied to determine the spatial resolution, yielding an average spatial resolution maintained at 24.5 ± 6 µm.

The ability to widen the optimised mass range to higher m/z values arises from the reduced velocities of heavier ions. These slower-moving ions can be more effectively separated and retained within the space between the extractor and the ion lens, allowing PEDA to operate effectively across an expanded mass window.

S2: Spatial resolution

The spatial resolution was determined by analysing the intensity profile across a grid image. A bold grid-line was selected, covering multiple pixels in width to improve statistics, with the spatial resolution defined as the distance across the intensity profile between the 20% and 80% levels. Additionally, a Gaussian fitting function could also be applied to the rising

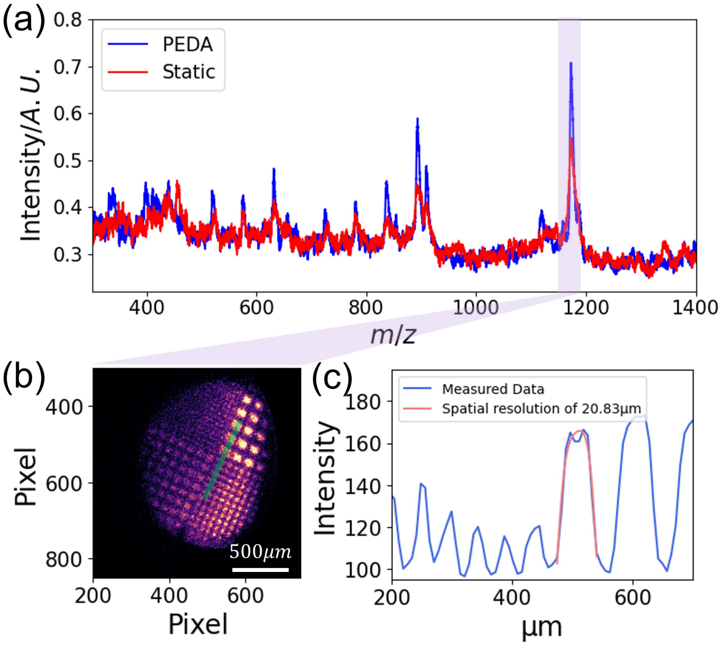

Figure S3: Panel (a) Comparison of Irganox 1010 mass spectra acquired with (blue) and without (red) PEDA. The data were obtained using a 450 ns trigger delay, using a MCP-P47 and PMT detector assembly. Panel (b) Irganox 1010 parent ion image obtained with PEDA. The grid pitch sizes range from 62 to 125 µm. Panel (c) Signal intensity profile of the selected region in (b), indicated by the green square. The light-red curve in (c) shows a Gaussian fit to the selected peak, which is used to determine the spatial resolution.

and falling edges of the intensity curve (see further below). The spatial resolution was then calculated using the full-width-at-half-maximum (FWHM), as given by:

$$\mathrm{FWHM} = 2\sqrt{2\ln 2}\,\sigma \approx 2.355\,\sigma\,, \tag{S1}$$

where σ is the standard deviation of the Gaussian.

As discussed in the main text, the spatial resolution of the instrument is limited by several factors. The magnification has a direct bearing on the measured spatial resolution of the ion image, as it determines the effective pixel size of the detector at the surface. Ion images of In^+ ions generated from SIMS of an ITO slide at different magnifications were shown in Figure 5 of the main text, and demonstrate the inverse relationship between spatial resolution and image magnification. A representative example of the associated (background)

mass spectrum of the ITO slide is shown in Figure S4.

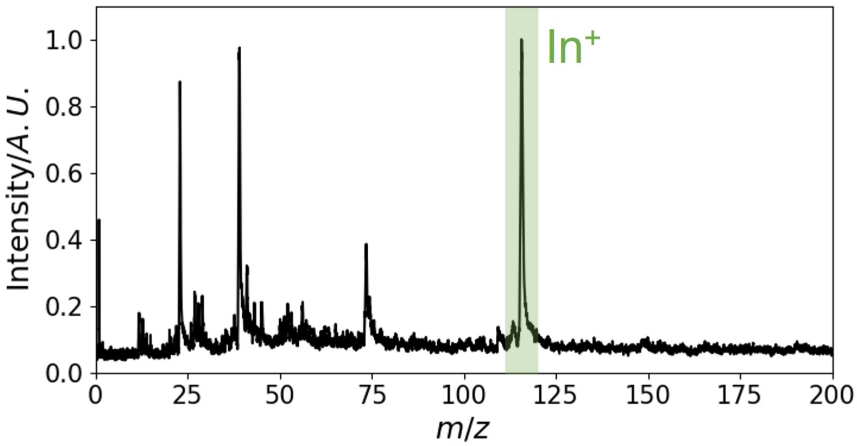

Figure S4: Mass spectrum of grid-patterned Rhodamine B on an ITO slide acquired with a static extraction field. The spectrum was collected using an MCP–P47 detector coupled to a photomultiplier tube (PMT). The In^+ signal, highlighted in green, was selected for spatial resolution measurements.

Another factor which can impact the spatial resolution is the size of a single ion event as recorded by the detector. Single ion events are typically detected by several pixels on the camera, which can therefore blur the spatial ion image. A centroiding process of single ion, multiple pixel events, is therefore essential to reduce the cluster of pixels to a single pixel and hence to enhance spatial resolution. Relevant centroiding algorithms have previously been implemented in ion imaging techniques.[3–6] Regarding spatial ion images, in this application the centroiding algorithm operates solely in 2D on the detector plane (see Figure S5). The position of each single ion event is recorded by the camera as a cluster of multiple pixels (x, y). For each ion event within a single camera frame, the algorithm identifies an initial single-pixel event and then searches the eight nearest neighboring pixels to group them into the same ion event cluster. This process is repeated for every pixel added to the cluster until no more adjacent pixels are found. Note that the number of pixels in the final centroided pixel array is the same as in uncentroided data.

Once all N pixels constituting a cluster have been identified, the centroid positions c_x and c_y are calculated as the intensity-weighted averages of the coordinates of each pixel in

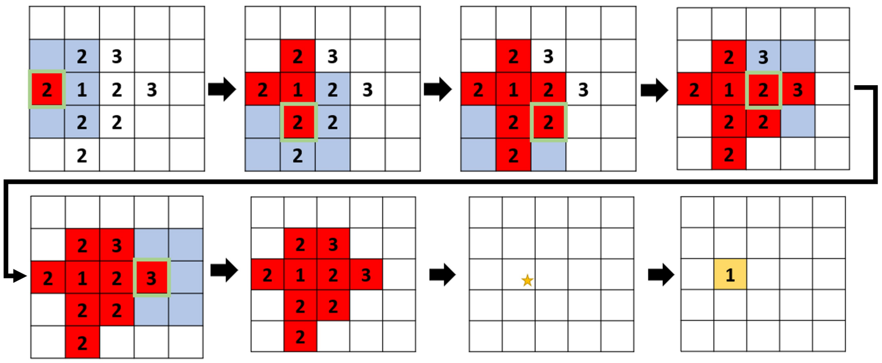

Figure S5: Schematic illustration of the centroiding of a single ion event in time and position. Each pixel within the cluster is labelled with its intensity value. Red pixels represent those initially identified as belonging to the cluster. Blue pixels indicate adjacent pixels that are examined during the search process; if an adjacent pixel has a non-zero intensity, it is added to the cluster, whereas a zero value terminates the search in that direction. Once all pixels belonging to the cluster have been identified, the centroid is calculated using the intensity-weighted positions of the pixels. The resulting centroided position is indicated by the yellow point.

the cluster:

$$\begin{aligned} c_x &= \sum_{i=1}^{N} x_i \cdot I_i \Big/ \sum_{i=1}^{N} I_i \\ c_y &= \sum_{i=1}^{N} y_i \cdot I_i \Big/ \sum_{i=1}^{N} I_i \end{aligned} \tag{S2}$$

Here, x_i and y_i represent the spatial coordinates of the ith pixel within the cluster, while I_i denotes the corresponding signal intensity. In the case of using the TimePix3-based camera, the time-over-threshold (ToT) feature was used as a surrogate for intensity.

The various analysis procedures to determine the spatial resolution are illustrated in Figure S6, in which a grid image was acquired using a CCD camera with a magnification of $\times 44.8$. For the uncentroided image shown in panel (a), using the conventional 20%–80% intensity method, the measured spatial resolution is 12.0 ± 1.6 µm. Using the Gaussian fitting method to the same image, the calculated spatial resolution is 13.0 ± 1.4 µm. After applying centroiding to the raw image (Figure S6 (c)), the centroided image yields a spatial

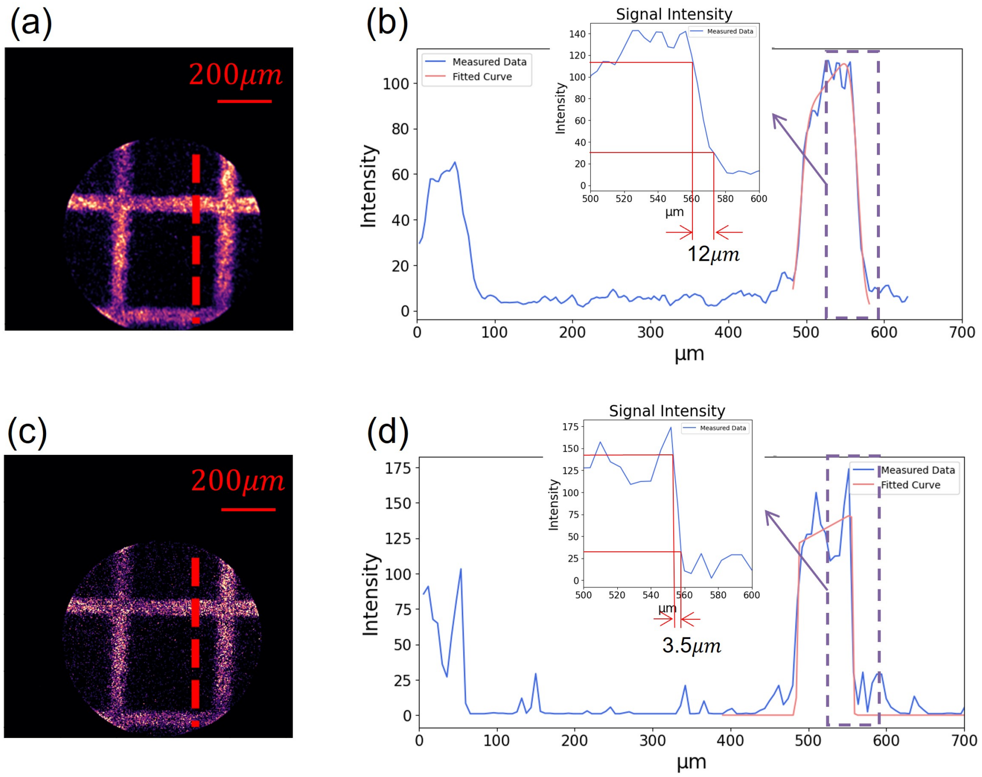

Figure S6: Panels (a) and (c) show the raw and centroided In^+ ion images of grided samples, respectively. The In^+ ions are generated directly from the ITO slide, and are visible in regions which have been masked by the wire grid structure during the dye electro-spraying process. Panels (b) and (d) display the corresponding intensity profiles, with Gaussian fitting applied to the edges of the peaks, as indicated by the light red lines on the blue profiles. The zoomed-in peak in the upper panels illustrates an alternative method for determining the spatial resolution, in which the distance between the 20% and 80% intensity points is used as the resolution metric.

resolution of $3.5 \pm 0.8\,\mu m$ employing the conventional analysis method, and $3.4 \pm 1.7\,\mu m$ using the Gaussian fitting method. The good agreement between both analysis approaches helps validate the derived spatial resolution data. Moreover, note that the centroiding process can enhance the spatial resolution by as much as a factor of four.

S3: Mass spectra for frozen mouse brain tissue

In the main text we demonstrate the application of the SIMS MSI instrument to imaging of a tissue sample. The mouse brain tissue employed was sampled at the infundibulum (IF)

and/or median eminence, with the imaging section selected to include regions spanning the fronto-parietal cortex (FrPT) and hippocampus (Hipp).[7] Panel (a) of Figure S7 illustrates the region of interest (a diameter of about 2 mm) within the brain, overlaid with an optical image to provide anatomical context. In this application, the PEDA pulse was optimised for a low mass range $m/z \leq 200$ using a trigger delay time of 180 ns (see Table 1 of the main text), and Panel (b) of Figure S7 presents the corresponding mass spectrum (m/z 0–700) of the sample.

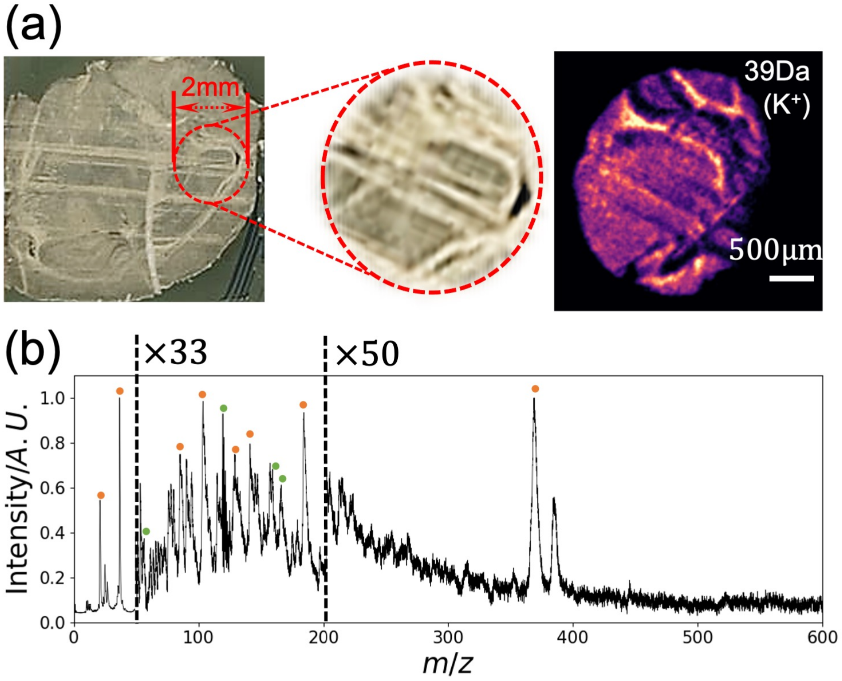

Figure S7: Panel (a) shows the optical image (left and middle panels) of the mouse brain tissue coronal section spanning the fronto-parietal cortex (FrPT) and hippocampus (Hipp). The zoomed in optical image is compared with the K^+ ion image shown in the right panel. Panel (b) shows the ToF mass spectrum of the mouse brain, collected using a MCP-P47-PMT system. Selected ion masses are indicated with orange markers and labeled in bold italic in Table S1; their corresponding spatial distributions are presented in Figure 6 of the main text. Ion masses indicated with green dots correspond to tryptophan fragment peaks at m/z 60, 120, and 159, while the peak at m/z 166 represents an additional phosphocholine fragment. The data were acquired using PEDA with a 180 ns trigger delay.

The resulting mass spectrum exhibits distinct ion peaks corresponding to biologically relevant species, including potassium (m/z 39, K^+), sodium (m/z 23, Na^+), and various

Table S1: Assignment of characteristic lipid fragment ions and their relation to parent molecules.[8–14] The ions highlighted in bold italic are those for which ion images are shown in the main text in Figure 6, and indicated with orange markers Figure S7.

Chemical formulae	Mass (m/z)	Parent molecule	Relevant to parent molecule
$C_2H_6NO^+$	60	Tryptophan	$[M–C_9H_6NO]^+$
$C_5H_{12}N^+$	86	Leucine	$[M–CHO_2]^+$
$C_5H_{14}NO^+$	104	Choline	M^+
$C_8H_{10}N^+$	120	Tryptophan	$[M–C_3H_2NO_2]^+$
$C_9H_8N^+$	130	Tryptophan	$[M–C_2H_4NO_2]^+$
$C_2H_8NO_4P^+$	141	Phosphatidylethanolamine	$[M–C_{37}H_{68}O_4]^+$
$C_{10}H_{11}N_2^+$	159	Tryptophan	$[M–CHO_2]^+$
$C_5H_{13}NO_3P^+$	166	Phosphocholine (PC)	$[M–H_2O]^+$
$C_5H_{15}NO_4P^+$	184	Phosphocholine (PC)	M^+
$C_{27}H_{45}^+$	369	Cholesterol	$[M–OH]^+$

lipid fragments (*e.g.*, m/z 184, $C_5H_{15}NO_4P^+$ and m/z 369, $C_{27}H_{45}^+$). Selected ion masses are indicated with orange markers and labeled in red in Table S1; their corresponding spatial distributions are presented in Figure 6 of the main text. Ion masses indicated with green dots correspond to tryptophan fragment peaks at m/z 60, 120, and 159, while the peak at m/z 166 represents an additional phosphocholine fragment.

S4: Simulations of possible instrument improvements

Based on our understanding of the microscope mode SIMS MSI instrument, it is clear that the physical distances between the repeller, extractor, and ion lens potentially play a role in determining the PEDA-optimised mass range, and the optimized mass and spatial resolution. To explore potential improvements in instrument performance, a series of simulations were conducted to inform the design of a next-generation instrument.

In the current configuration, the distance between the repeller and extractor ion optics is 5.5 mm, and that between the repeller and ion lens is 12 mm, as shown in Figure S8 (a). Initially, the ion lens assembly was repositioned further from the extractor, increasing the repeller-to-lens distance to 17 mm. As shown in Figures S8(b) and (b), this modification broadened the optimised mass range from m/z 250–600 to m/z 250–750, due to an expanded

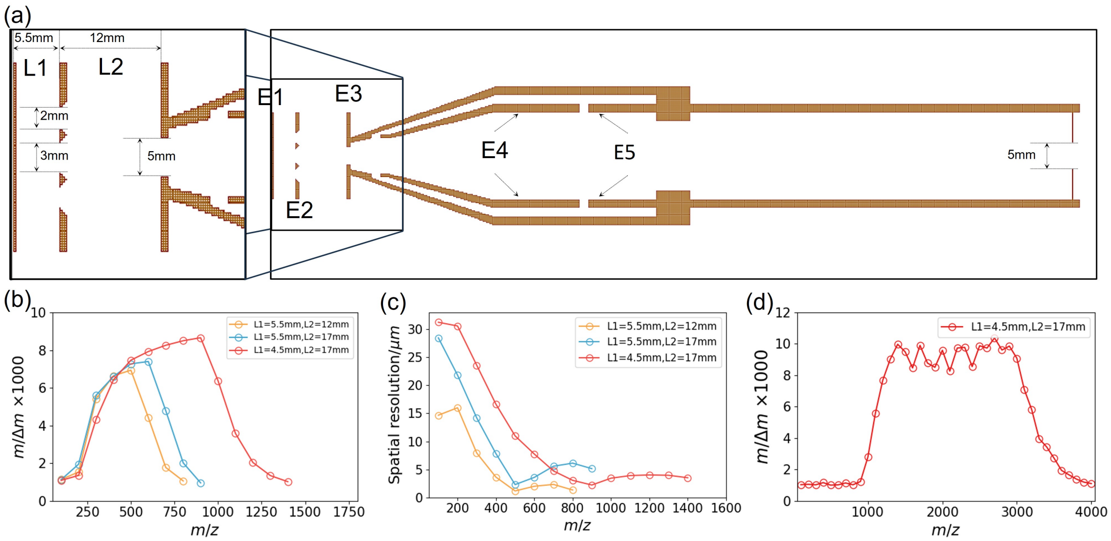

Figure S8: (a) SIMION ion optics model of the current design, with a 5.5 mm repeller–extractor gap and a 12 mm repeller–lens distance. (b) and (c) show the mass resolution and spatial resolution over the optimised mass range after modifying the lens distance. (d) Simulation of mass resolution for optimised detection above m/z 1000. In all cases, the simulations employed +8.0 kV on the repeller stage, and 5.5 kV on the extractor (see Table S2 for a full list of PEDA settings).

region for ion storage between the extractor and lens.

Table S2: Settings for different time-variable PEDA configurations used in conjunction with the simulations shown in Figure S8. The voltages (in Volts) correspond to the repeller voltage (V_R), the extractor baseline voltage (V_E), the first extractor pulse voltage (V_1), the second pulse voltage (V_2), and the lens voltage (V_L). The parameter t_d defines the trigger delay time (in nanoseconds) of the PEDA pulse.

Setting	t_d	V_R	V_E	V_1	V_2	V_L
PEDA (L1 5.5 mm, L2 12 mm)	380	8000	5530	5870	6870	−15400
PEDA (L1 5.5 mm, L2 17 mm)	420	8000	5530	5980	7780	−14100
PEDA (L1 4.5 mm, L2 17 mm)	420	8000	5530	5980	8380	−14800
PEDA (L1 4.5 mm, L2 17 mm)	800	8000	5530	5940	7940	−14500

Subsequently, by maintaining the increased extractor-to-lens distance at 17 mm and reducing the repeller–extractor gap from 5.5 mm to 4.5 mm, the optimised mass range was

further extended to m/z 250–1100, nearly doubling that of the original configuration (see Figures S8(b) and (c)). This improvement results from the ions being more rapidly accelerated, which compresses the mass clusters and reduces their spatial dispersion prior to entering the extractor–lens region, thereby enhancing the effectiveness of PEDA.

For even higher mass species ($m/z \gtrsim 1000$), simulations indicate that the optimised mass range can reach up to $m/z \sim 2200$ by changing the PEDA trigger time from 420 ns to 800 ns in the simulation, as shown in Figure S8(d). Such a mass range is suitable for the detection of most lipids and metabolites. It seems likely that further improvements to specific aspects of instrument performance could be achieved by more significant changes to the ion optic design, not explored in the current work.

References

(1) Guo, A.; Burt, M.; Brouard, M. Mass-resolved ion microscope imaging over expanded mass ranges using double-field post-extraction differential acceleration. *Int. J. Mass Spectrom.* **2018**, *429*, 121–126.

(2) Guo, A.; Burleigh, R. J.; Smith, N.; Brouard, M.; Burt, M. High-Resolution Ion Microscope Imaging over Wide Mass Ranges Using Electrodynamic Postextraction Differential Acceleration. *J. Am. Soc. Mass Spectrom.* **2020**, *31*, 1903–1909.

(3) Halford, E.; Winter, B.; Mills, M. D.; Thompson, S. P.; Parr, V.; John, J. J.; Nomerotski, A.; Vallance, C.; Turchetta, R.; Brouard, M. Modifications to a commercially available linear mass spectrometer for mass-resolved microscopy with the pixel imaging mass spectrometry (PImMS) camera. *Rapid Commun. Mass Spectrom.* **2014**, *28*, 1649–1657.

(4) Slater, C. S.; Blake, S.; Brouard, M.; Lauer, A.; Vallance, C.; John, J. J.; Turchetta, R.; Nomerotski, A.; Christensen, L.; Nielsen, J. H.; Johansson, M. P.; Stapelfeldt, H. Co-

variance imaging experiments using a pixel-imaging mass-spectrometry camera. *Phys. Rev. A* **2014**, *89*, 011401.

(5) Zhao, A.; van Beuzekom, M.; Bouwens, B.; Byelov, D.; Chakaberia, I.; Cheng, C.; Maddox, E.; Nomerotski, A.; Svihra, P.; Visser, J.; Vrba, V.; Weinacht, T. Coincidence velocity map imaging using Tpx3Cam, a time stamping optical camera with 1.5 ns timing resolution. *Rev. Sci. Instrum.* **2017**, *88*, 113104.

(6) Jia, Y.; Green, F. M.; Cheung, K.; Castellani, M. E.; Brouard, M. Multimass Three-Dimensional Velocity Map Imaging from Surfaces. *J Phys. Chem. Lett.* **2025**, *16*, 11762.

(7) Rao, D. B.; Little, P. B.; Sills, R. C. Subsite Awareness in Neuropathology Evaluation of National Toxicology Program (NTP) Studies: A Review of Select Neuroanatomical Structures with Their Functional Significance in Rodents. *Tox. Path.* **2014**, *42*, 487–509.

(8) Siegel, G. J. *Basic neurochemistry: molecular, cellular, and medical aspects*, 6th ed.; Lippincott Williams & Wilkins, Philadelphia, USA, 1999.

(9) Pulfer, M.; Murphy, R. C. Electrospray mass spectrometry of phospholipids. *Mass Spectrom. Rev.* **2003**, *22*, 332–364.

(10) Sjövall, P.; Lausmaa, J.; Johansson, B. Mass Spectrometric Imaging of Lipids in Brain Tissue. *Anal. Chem.* **2004**, *76*, 4271–4278.

(11) Xia, Y.-Q.; Jemal, M. Phospholipids in liquid chromatography/mass spectrometry bioanalysis: comparison of three tandem mass spectrometric techniques for monitoring plasma phospholipids, the effect of mobile phase composition on phospholipids elution and the association of phospholipids with matrix effects. *Rapid Commun. Mass Spectrom.* **2009**, *23*, 2125–2138.

(12) Lanni, E. J.; Dunham, S. J. B.; Nemes, P.; Rubakhin, S. S.; Sweedler, J. V. Biomolecular Imaging with a C_{60}-SIMS/MALDI Dual Ion Source Hybrid Mass Spectrometer: Instrumentation, Matrix Enhancement, and Single Cell Analysis. *J. Am. Soc. Mass Spectrom.* **2014**, *25*, 1897–1907.

(13) Rakowska, P. D.; Seah, M. P.; Vorng, J.-L.; Havelund, R.; Gilmore, I. S. Determination of the sputtering yield of cholesterol using Ar_n^+ and $C_{60}^{+(+)}$ cluster ions. *Analyst* **2016**, *141*, 4893–4901.

(14) Suvannapruk, W.; Edney, M. K.; Kim, D.-H.; Scurr, D. J.; Ghaemmaghami, A. M.; Alexander, M. R. Single-Cell Metabolic Profiling of Macrophages Using 3D OrbiSIMS: Correlations with Phenotype. *Anal. Chem.* **2022**, *94*, 9389–9398.
